# Supplementary material for: DeepCristae, a CNN for the restoration of mitochondria cristae in live microscopy images
Source: Commun Biol. 2025 Feb 26;8:320. doi: 10.1038/s42003-025-07684-x (PMC11865493; doi:10.1038/s42003-025-07684-x)
Supplement: Supplementary file 3 — Description of Additional Supplementary Files [file 42003_2025_7684_MOESM3_ESM.pdf]

Description of Additional Supplementary Files

File name: Supplementary Movie 1

Title: DeepCristae restoration enhances cristae resolution in live imaging using a Spinning-Disk confocal microscope equipped with Live-SR (related to Fig. 7e).

Description: 3D stacks of live RPE1 cells labeled with PKMITO-Orange (also known as PKMO) were acquired with a Spinning-Disk confocal microscope equipped with a Super Resolution module. Mitochondrial fission events are shown.

File name: Supplementary Movie 2

Title: DeepCristae restoration enhances cristae resolution in live imaging using a Lattice Light Sheet microscope (related to Fig. 7f).

Description: 3D stacks of live RPE1 cells labeled with PKMITO-Orange (also known as PKMO) were acquired with a Lattice Light Sheet Microscope (denoted LLSM). Fusion and fission dynamics of mitochondria are indicated in a zoomed region.

File name: Supplementary Movie 3

Title: DeepCristae reveals 3D+time cristae morphology during endo/lysosome mitochondria interactions using a Spinning-Disk confocal microscope equipped with Live-SR (related to Fig. 8a).

Description: RPE1 cells incubated with CellMask Plasma Membrane Deep Red (red) (denoted PMDR) were labeled with PKMITO-Orange (also known as PKMO) (green). 3D stacks were acquired every 1.86 s per channel with Live-SR microscopy. Data is shown before and after DeepCristae restoration of the mitochondria (green channel) as well as after denoising (ND-SAFIR) and RichardsonLucy deconvolution of the endo/lysosomes (red channel). Insets correspond to Fig. 8a.

File name: Supplementary Movie 4

Title: DeepCristae reveals 3D+time cristae morphology during endo/lysosome mitochondria interactions using Lattice Light Sheet microscope (related to Supplementary Fig. 5d-e).

Description: RPE1 cells incubated with CellMask Plasma Membrane Deep Red (denoted PMDR) (red) were labeled with PKMITO-Orange (also known as PKMO) (green). 3D stacks were acquired every 0.49 s per channel with Lattice Light Sheet Microscopy (denoted LLSM). Data is shown after deskewing and Richardson-Lucy deconvolution before and after DeepCristae restoration of the mitochondria (green channel) as well as after denoising (ND-SAFIR) of the endo/lysosomes (red channel). Insets correspond to Supplementary Fig. 5d.
